# 4,4′,6,6′-Tetra­bromo-2,2′-(2,8-diazonia-5-azanona-1,8-diene-1,9-diyl)diphenolate

**DOI:** 10.1107/S1600536808037732

**Published:** 2008-11-26

**Authors:** Zhu-Jun Chen, Ke-Wei Lei

**Affiliations:** a Zhejiang Textile and Fashion College, Ningbo 315211, People’s Republic of China; b State Key Laboratory Base of Novel Functional Materials and Preparation Science, Institute of Solid Materials Chemistry, Faculty of Materials Science and Chemical Engineering, Ningbo University, Ningbo 315211, People’s Republic of China

## Abstract

In the zwitterionic title compound, C_18_H_17_Br_4_N_3_O_2_, the two salicylaldimine groups form a dihedral angle of 51.94 (2)° and the dihedral angle between the aromatic ring planes is 51.14 (2)°. One of the C atoms adjacent to the aza N atom is disordered over two positions; the site-occupancy factors are 0.51 (1) and 0.49 (1). There are two strong intra­molecular N—H⋯O hydrogen bonds in the mol­ecule.

## Related literature

For general background on the use of Schiff bases in metal complexes, see: Vigato *et al.* (2007 ▶).
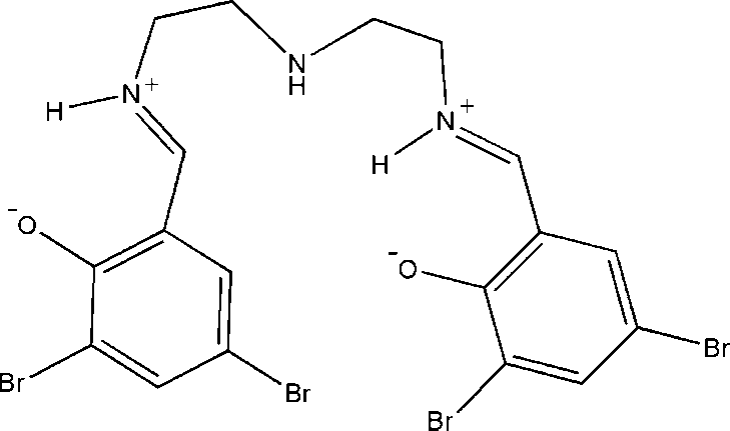


## Experimental

### 

#### Crystal data

C_18_H_17_Br_4_N_3_O_2_
*M* *_r_* = 626.99
Monoclinic, 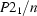

*a* = 9.4506 (11) Å
*b* = 9.1242 (11) Å
*c* = 23.618 (3) Å
β = 94.774 (2)°
*V* = 2029.5 (4) Å^3^
*Z* = 4
Mo *K*α radiation
μ = 7.95 mm^−1^
*T* = 293 (2) K
0.26 × 0.21 × 0.19 mm

#### Data collection

Bruker SMART APEXII diffractometer
Absorption correction: multi-scan (*SADABS*; Sheldrick, 1997 ▶) *T*_min_ = 0.149, *T*_max_ = 0.227
17118 measured reflections
4693 independent reflections
3747 reflections with *I* > 2σ(*I*)
*R*_int_ = 0.040

#### Refinement

*R*[*F* ^2^ > 2σ(*F* ^2^)] = 0.038
*wR*(*F* ^2^) = 0.095
*S* = 1.05
4693 reflections
256 parameters
6 restraints
H atoms treated by a mixture of independent and constrained refinement
Δρ_max_ = 2.07 e Å^−3^
Δρ_min_ = −0.94 e Å^−3^

### 

Data collection: *APEX2* (Bruker, 2004 ▶); cell refinement: *SAINT* (Bruker, 2004 ▶); data reduction: *SAINT*; program(s) used to solve structure: *SHELXS97* (Sheldrick, 2008 ▶); program(s) used to refine structure: *SHELXL97* (Sheldrick, 2008 ▶); molecular graphics: *XP* in *SHELXTL* (Sheldrick, 2008 ▶); software used to prepare material for publication: *XP* in *SHELXTL*.

## Supplementary Material

Crystal structure: contains datablocks global, I. DOI: 10.1107/S1600536808037732/bq2108sup1.cif

Structure factors: contains datablocks I. DOI: 10.1107/S1600536808037732/bq2108Isup2.hkl

Additional supplementary materials:  crystallographic information; 3D view; checkCIF report

## Figures and Tables

**Table 1:** Hydrogen-bond geometry (Å, °)

*D*—H⋯*A*	*D*—H	H⋯*A*	*D*⋯*A*	*D*—H⋯*A*
N1—H1*A*⋯O1	0.97 (6)	1.70 (6)	2.553 (5)	144 (5)
N3—H3*A*⋯O2	0.87 (6)	1.84 (6)	2.597 (4)	144 (5)

**Fig. 1.**
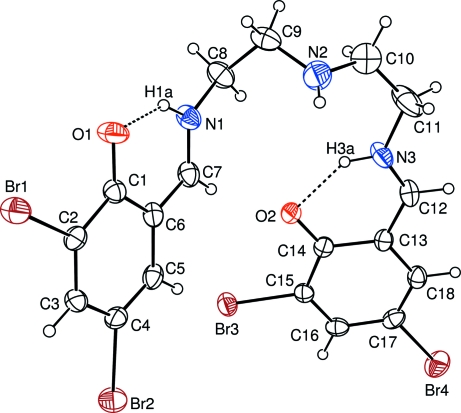
The structure of (I), showing 50% probability displacement ellipsoids and the atom-numbering scheme. Dashed lines show H-bondings. Only the major component is shown.

## supplementary crystallographic information

### Comment

The Schiff bases are widely employed as ligands in coordination chemistry. These ligands are readily available, versatile and, depending on the nature of the starting materials (primary amines and carbonyl precursors), they exhibit various denticities and functionalities. Moreover, the number, the nature, and the relative position of the donor atoms of a Schiff base ligand allow a good control over the stereochemistry of the metallic centers, as well as over the number of the metal ions within homo- and heteropolynuclear complexes. All these advantages make Schiff bases very good candidates in the effort to synthesize metal complexes of interest in bioinorganic chemistry, catalysis, encapsulation, transport and separation processes, magnetochemistry (Vigato *et al.*, 2007). So we report here the crystal structure of the new Schiff base ligand, 4,4',6,6'-Tetrabromo-2,2'-[3-azapentane- 1,5-diylbis(nitrilomethylidyne)]diphenol(I).

The molecular structure of (I) is illustrated in Fig. 1. The two pendant moieties in a *cis* conformation attach to the ends of the C—C—N—C—C backbone. The N2 atom exhibits tetrahedral *sp*^3^ hybridization, whereas the two amide N atoms display planar *sp*^2^ hybridization. There is no H atom attached to O1 and O2 atoms. Instead these H atoms are attached to the N1 and N3 atoms. The double-bonds C7—N1 (1.295 (6) Å) and C12—N3 (1.296 (6) Å) show the typical character of Schiff base. The dihedral angle between the salicylaldimine groups is 51.94 (2)°. The crystal structure of (I) is stabilized by intramolecular N—H···O hydrogen bonding. The C10 atom is disorder over two positions with the site-occupancy factors of 0.51 (1) and 0.49 (1). The larger than normal range of thermal motion is mostly due to the difference between the disordered group and the other atoms which are not disordered.

### Experimental

*N*-(2-aminoethyl)ethane-1,2-diamine (0.01 mol, 1.03 g) and 2-hydroxy-3,5-dibromobenzaldehyde(0.02 mol, 5.60 g) were dissolved in 20 ml e thanol and the solution was stirred for 3 h. After filtration and evaporation, a pure yellow product was recrystallized from ethanol. Yield: 81.7%. Calcd. for C_18_H_17_Br_4_N_3_O_2_: C, 34.48; H, 2.73; N, 6.70; Found: C, 34.59; H, 2.62; N, 6.81%.

### Refinement

All H atoms except the N attached H1A and H3A which refined freely were placed in geometrically idealized positions and constrained to ride on their parent atoms (C—H = 0.93%A, 0.97%A; N—H = 0.86 Å; and *U*_iso_(H) values equal to 1.2 *U*_eq_C.

### Crystal data

**Table:**

C_18_H_17_Br_4_N_3_O_2_	*F*_000_ = 1208
*M**_r_* = 626.99	*D*_x_ = 2.052 Mg m^−^^3^
Monoclinic, *P*2_1_/*n*	Mo *K*α radiation λ = 0.71073 Å
Hall symbol: -P 2yn	Cell parameters from 5793 reflections
*a* = 9.4506 (11) Å	θ = 1.0–27.6º
*b* = 9.1242 (11) Å	µ = 7.95 mm^−^^1^
*c* = 23.618 (3) Å	*T* = 293 (2) K
β = 94.774 (2)º	BLOCK, yellow
*V* = 2029.5 (4) Å^3^	0.26 × 0.21 × 0.19 mm
*Z* = 4	

### Data collection

**Table:**

Bruker SMART APEXII diffractometer	4693 independent reflections
Radiation source: fine-focus sealed tube	3747 reflections with *I* > 2σ(*I*)
Monochromator: graphite	*R*_int_ = 0.040
*T* = 293(2) K	θ_max_ = 27.6º
φ and ω scans	θ_min_ = 1.7º
Absorption correction: multi-scan(SADABS; Sheldrick, 1997)	*h* = −12→12
*T*_min_ = 0.149, *T*_max_ = 0.227	*k* = −11→10
17118 measured reflections	*l* = −30→30

### Refinement

**Table:**

Refinement on *F*^2^	Secondary atom site location: difference Fourier map
Least-squares matrix: full	Hydrogen site location: inferred from neighbouring sites
*R*[*F*^2^ > 2σ(*F*^2^)] = 0.038	H atoms treated by a mixture of independent and constrained refinement
*wR*(*F*^2^) = 0.095	*w* = 1/[σ^2^(*F*_o_^2^) + (0.0444*P*)^2^ + 3.6177*P*] where *P* = (*F*_o_^2^ + 2*F*_c_^2^)/3
*S* = 1.05	(Δ/σ)_max_ = 0.001
4693 reflections	Δρ_max_ = 2.07 e Å^−^^3^
256 parameters	Δρ_min_ = −0.94 e Å^−^^3^
6 restraints	Extinction correction: none
Primary atom site location: structure-invariant direct methods	

### Special details

**Table:**

Geometry. All e.s.d.'s (except the e.s.d. in the dihedral angle between two l.s. planes) are estimated using the full covariance matrix. The cell e.s.d.'s are taken into account individually in the estimation of e.s.d.'s in distances, angles and torsion angles; correlations between e.s.d.'s in cell parameters are only used when they are defined by crystal symmetry. An approximate (isotropic) treatment of cell e.s.d.'s is used for estimating e.s.d.'s involving l.s. planes.
Refinement. Refinement of *F*^2^ against ALL reflections. The weighted *R*-factor *wR* and goodness of fit *S* are based on *F*^2^, conventional *R*-factors *R* are based on *F*, with *F* set to zero for negative *F*^2^. The threshold expression of *F*^2^ > σ(*F*^2^) is used only for calculating *R*-factors(gt) *etc*. and is not relevant to the choice of reflections for refinement. *R*-factors based on *F*^2^ are statistically about twice as large as those based on *F*, and *R*- factors based on ALL data will be even larger.

### Fractional atomic coordinates and isotropic or equivalent isotropic displacement parameters (Å^2^)

**Table:**

	*x*	*y*	*z*	*U*_iso_*/*U*_eq_	Occ. (<1)
C1	1.1639 (4)	1.1651 (4)	0.03605 (16)	0.0255 (8)	
C2	1.2507 (4)	1.0675 (4)	0.00631 (15)	0.0244 (8)	
C3	1.2518 (4)	0.9186 (4)	0.01388 (16)	0.0260 (8)	
H3	1.3097	0.8596	−0.0065	0.031*	
C4	1.1645 (4)	0.8557 (4)	0.05281 (18)	0.0280 (9)	
C5	1.0778 (4)	0.9402 (5)	0.08246 (17)	0.0277 (8)	
H5	1.0197	0.8968	0.1076	0.033*	
C6	1.0763 (4)	1.0945 (5)	0.07503 (16)	0.0252 (8)	
C7	0.9904 (4)	1.1819 (5)	0.10911 (17)	0.0286 (9)	
H7	0.9341	1.1363	0.1344	0.034*	
C8	0.9087 (5)	1.4217 (5)	0.13912 (18)	0.0362 (10)	
H8A	0.9734	1.4858	0.1615	0.043*	
H8B	0.8566	1.3647	0.1651	0.043*	
C9	0.8055 (5)	1.5135 (5)	0.10130 (19)	0.0367 (10)	
H9A	0.7693	1.5925	0.1235	0.044*	
H9B	0.8551	1.5567	0.0711	0.044*	
C10	0.5604 (11)	1.4631 (12)	0.1098 (5)	0.0500 (18)	0.501 (9)
H10A	0.5928	1.4777	0.1495	0.060*	0.501 (9)
H10B	0.5188	1.5544	0.0955	0.060*	0.501 (9)
C10'	0.5427 (11)	1.4357 (13)	0.0666 (5)	0.0500 (18)	0.499 (9)
H10C	0.5168	1.3988	0.0286	0.060*	0.499 (9)
H10D	0.5160	1.5383	0.0670	0.060*	0.499 (9)
C11	0.4590 (6)	1.3573 (6)	0.1061 (3)	0.0619 (17)	
H11A	0.4238	1.3449	0.0666	0.074*	
H11B	0.3801	1.3881	0.1270	0.074*	
C12	0.4349 (4)	1.1126 (5)	0.14599 (17)	0.0303 (9)	
H12	0.3368	1.1242	0.1417	0.036*	
C13	0.4899 (4)	0.9821 (4)	0.17117 (15)	0.0235 (8)	
C14	0.6423 (4)	0.9637 (4)	0.18085 (15)	0.0228 (8)	
C15	0.6853 (4)	0.8296 (4)	0.20880 (15)	0.0227 (8)	
C16	0.5925 (4)	0.7256 (4)	0.22511 (16)	0.0259 (8)	
H16	0.6260	0.6405	0.2433	0.031*	
C17	0.4452 (4)	0.7494 (4)	0.21399 (17)	0.0277 (8)	
C18	0.3950 (4)	0.8739 (5)	0.18751 (16)	0.0266 (8)	
H18	0.2977	0.8875	0.1802	0.032*	
Br1	1.37074 (5)	1.15131 (5)	−0.045130 (17)	0.03273 (12)	
Br2	1.17523 (5)	0.64919 (5)	0.06248 (2)	0.04388 (14)	
Br3	0.88370 (4)	0.79812 (5)	0.222670 (17)	0.03065 (12)	
Br4	0.31565 (5)	0.60611 (6)	0.23712 (2)	0.04384 (14)	
N1	0.9899 (4)	1.3230 (4)	0.10528 (15)	0.0310 (8)	
N2	0.6880 (4)	1.4267 (5)	0.07679 (18)	0.0447 (10)	
H2	0.6896	1.3661	0.0490	0.054*	
N3	0.5128 (4)	1.2170 (4)	0.12852 (16)	0.0342 (8)	
O1	1.1653 (3)	1.3038 (3)	0.02905 (13)	0.0365 (7)	
O2	0.7294 (3)	1.0603 (3)	0.16669 (11)	0.0265 (6)	
H1A	1.050 (6)	1.358 (6)	0.077 (2)	0.052 (15)*	
H3A	0.603 (6)	1.199 (6)	0.137 (2)	0.052 (16)*	

### Atomic displacement parameters (Å^2^)

**Table:**

	*U*^11^	*U*^22^	*U*^33^	*U*^12^	*U*^13^	*U*^23^
C1	0.0248 (19)	0.026 (2)	0.0259 (19)	0.0012 (16)	0.0033 (14)	0.0018 (15)
C2	0.0241 (19)	0.026 (2)	0.0231 (18)	−0.0036 (15)	0.0034 (14)	0.0021 (15)
C3	0.0238 (19)	0.024 (2)	0.0295 (19)	0.0033 (15)	−0.0026 (15)	−0.0002 (16)
C4	0.025 (2)	0.021 (2)	0.037 (2)	−0.0015 (16)	−0.0051 (16)	0.0068 (17)
C5	0.0217 (19)	0.030 (2)	0.031 (2)	−0.0039 (16)	−0.0001 (15)	0.0095 (17)
C6	0.0200 (18)	0.030 (2)	0.0259 (19)	0.0013 (16)	0.0020 (14)	0.0043 (16)
C7	0.024 (2)	0.033 (2)	0.029 (2)	0.0017 (17)	0.0043 (15)	0.0062 (17)
C8	0.040 (2)	0.038 (3)	0.032 (2)	0.009 (2)	0.0082 (18)	−0.0018 (19)
C9	0.047 (3)	0.028 (2)	0.037 (2)	0.009 (2)	0.0102 (19)	−0.0003 (18)
C10	0.050 (4)	0.050 (4)	0.050 (4)	0.000 (3)	0.004 (4)	0.000 (4)
C10'	0.050 (4)	0.050 (4)	0.050 (4)	0.000 (3)	0.004 (4)	0.000 (4)
C11	0.046 (3)	0.049 (3)	0.094 (5)	0.021 (3)	0.026 (3)	0.042 (3)
C12	0.0202 (19)	0.041 (3)	0.030 (2)	0.0029 (17)	0.0028 (15)	0.0027 (18)
C13	0.0210 (18)	0.029 (2)	0.0210 (17)	−0.0010 (15)	0.0009 (14)	−0.0005 (15)
C14	0.0226 (18)	0.027 (2)	0.0194 (17)	−0.0007 (16)	0.0040 (14)	−0.0058 (15)
C15	0.0234 (18)	0.024 (2)	0.0205 (17)	0.0015 (15)	0.0031 (14)	−0.0046 (15)
C16	0.034 (2)	0.022 (2)	0.0232 (18)	0.0001 (16)	0.0073 (15)	−0.0017 (15)
C17	0.030 (2)	0.027 (2)	0.0271 (19)	−0.0079 (17)	0.0100 (15)	−0.0036 (16)
C18	0.0217 (19)	0.033 (2)	0.0249 (18)	0.0004 (16)	0.0036 (14)	−0.0046 (16)
Br1	0.0407 (2)	0.0288 (2)	0.0309 (2)	0.00118 (18)	0.01642 (17)	0.00159 (16)
Br2	0.0412 (3)	0.0226 (2)	0.0680 (3)	0.00063 (19)	0.0055 (2)	0.0134 (2)
Br3	0.0242 (2)	0.0332 (2)	0.0343 (2)	0.00332 (17)	0.00105 (15)	0.00357 (17)
Br4	0.0378 (3)	0.0422 (3)	0.0531 (3)	−0.0142 (2)	0.0130 (2)	0.0056 (2)
N1	0.0311 (19)	0.031 (2)	0.0321 (18)	0.0054 (15)	0.0115 (15)	0.0043 (15)
N2	0.040 (2)	0.042 (3)	0.052 (2)	−0.0019 (19)	0.0068 (18)	0.0108 (19)
N3	0.0275 (19)	0.035 (2)	0.040 (2)	0.0112 (16)	0.0045 (15)	0.0110 (17)
O1	0.0476 (19)	0.0202 (15)	0.0448 (17)	0.0028 (14)	0.0220 (14)	0.0030 (13)
O2	0.0227 (13)	0.0244 (15)	0.0325 (14)	−0.0013 (11)	0.0037 (11)	0.0024 (11)

### Geometric parameters (Å, °)

**Table:**

C1—O1	1.277 (5)	C10'—N2	1.376 (11)
C1—C2	1.434 (6)	C10'—C11	1.459 (12)
C1—C6	1.440 (5)	C10'—H10C	0.9700
C2—C3	1.370 (6)	C10'—H10D	0.9700
C2—Br1	1.892 (4)	C11—N3	1.460 (6)
C3—C4	1.408 (6)	C11—H11A	0.9700
C3—H3	0.9300	C11—H11B	0.9700
C4—C5	1.360 (6)	C12—N3	1.293 (6)
C4—Br2	1.900 (4)	C12—C13	1.411 (6)
C5—C6	1.419 (6)	C12—H12	0.9300
C5—H5	0.9300	C13—C18	1.409 (6)
C6—C7	1.433 (6)	C13—C14	1.449 (5)
C7—N1	1.290 (6)	C14—O2	1.270 (5)
C7—H7	0.9300	C14—C15	1.433 (5)
C8—N1	1.463 (5)	C15—C16	1.369 (6)
C8—C9	1.518 (6)	C15—Br3	1.898 (4)
C8—H8A	0.9700	C16—C17	1.412 (6)
C8—H8B	0.9700	C16—H16	0.9300
C9—N2	1.445 (6)	C17—C18	1.363 (6)
C9—H9A	0.9700	C17—Br4	1.902 (4)
C9—H9B	0.9700	C18—H18	0.9300
C10—C11	1.359 (12)	N1—H1A	0.97 (6)
C10—N2	1.526 (11)	N2—H2	0.8600
C10—H10A	0.9700	N3—H3A	0.87 (6)
C10—H10B	0.9700		
			
O1—C1—C2	122.7 (4)	H10C—C10'—H10D	107.3
O1—C1—C6	122.6 (4)	C10—C11—C10'	43.6 (6)
C2—C1—C6	114.7 (4)	C10—C11—N3	112.1 (7)
C3—C2—C1	123.4 (4)	C10'—C11—N3	118.1 (6)
C3—C2—Br1	119.1 (3)	C10—C11—H11A	109.2
C1—C2—Br1	117.5 (3)	C10'—C11—H11A	66.7
C2—C3—C4	119.4 (4)	N3—C11—H11A	109.2
C2—C3—H3	120.3	C10—C11—H11B	109.2
C4—C3—H3	120.3	C10'—C11—H11B	131.6
C5—C4—C3	121.0 (4)	N3—C11—H11B	109.2
C5—C4—Br2	121.9 (3)	H11A—C11—H11B	107.9
C3—C4—Br2	117.0 (3)	N3—C12—C13	123.8 (4)
C4—C5—C6	119.9 (4)	N3—C12—H12	118.1
C4—C5—H5	120.0	C13—C12—H12	118.1
C6—C5—H5	120.0	C18—C13—C12	119.1 (3)
C5—C6—C7	118.9 (4)	C18—C13—C14	121.5 (4)
C5—C6—C1	121.5 (4)	C12—C13—C14	119.4 (4)
C7—C6—C1	119.5 (4)	O2—C14—C15	123.3 (3)
N1—C7—C6	121.0 (4)	O2—C14—C13	122.4 (4)
N1—C7—H7	119.5	C15—C14—C13	114.3 (3)
C6—C7—H7	119.5	C16—C15—C14	123.9 (4)
N1—C8—C9	111.0 (4)	C16—C15—Br3	119.6 (3)
N1—C8—H8A	109.4	C14—C15—Br3	116.5 (3)
C9—C8—H8A	109.4	C15—C16—C17	119.1 (4)
N1—C8—H8B	109.4	C15—C16—H16	120.5
C9—C8—H8B	109.4	C17—C16—H16	120.5
H8A—C8—H8B	108.0	C18—C17—C16	120.9 (4)
N2—C9—C8	111.6 (4)	C18—C17—Br4	119.8 (3)
N2—C9—H9A	109.3	C16—C17—Br4	119.3 (3)
C8—C9—H9A	109.3	C17—C18—C13	120.3 (4)
N2—C9—H9B	109.3	C17—C18—H18	119.8
C8—C9—H9B	109.3	C13—C18—H18	119.8
H9A—C9—H9B	108.0	C7—N1—C8	125.1 (4)
C11—C10—N2	113.3 (8)	C7—N1—H1A	112 (3)
C11—C10—H10A	108.9	C8—N1—H1A	123 (3)
N2—C10—H10A	108.9	C10'—N2—C9	139.4 (6)
C11—C10—H10B	108.9	C10'—N2—C10	42.1 (6)
N2—C10—H10B	108.9	C9—N2—C10	106.8 (5)
H10A—C10—H10B	107.7	C10'—N2—H2	89.2
N2—C10'—C11	116.4 (8)	C9—N2—H2	126.6
N2—C10'—H10C	108.2	C10—N2—H2	126.6
C11—C10'—H10C	108.2	C12—N3—C11	124.8 (4)
N2—C10'—H10D	108.2	C12—N3—H3A	111 (4)
C11—C10'—H10D	108.2	C11—N3—H3A	123 (4)
			
O1—C1—C2—C3	179.1 (4)	C12—C13—C14—O2	−1.1 (6)
C6—C1—C2—C3	−0.1 (6)	C18—C13—C14—C15	−1.0 (5)
O1—C1—C2—Br1	−0.1 (5)	C12—C13—C14—C15	177.5 (3)
C6—C1—C2—Br1	−179.3 (3)	O2—C14—C15—C16	178.9 (4)
C1—C2—C3—C4	−0.3 (6)	C13—C14—C15—C16	0.3 (5)
Br1—C2—C3—C4	178.9 (3)	O2—C14—C15—Br3	−1.6 (5)
C2—C3—C4—C5	0.8 (6)	C13—C14—C15—Br3	179.8 (3)
C2—C3—C4—Br2	−178.4 (3)	C14—C15—C16—C17	0.2 (6)
C3—C4—C5—C6	−1.0 (6)	Br3—C15—C16—C17	−179.3 (3)
Br2—C4—C5—C6	178.2 (3)	C15—C16—C17—C18	−0.1 (6)
C4—C5—C6—C7	−176.5 (4)	C15—C16—C17—Br4	−179.5 (3)
C4—C5—C6—C1	0.7 (6)	C16—C17—C18—C13	−0.6 (6)
O1—C1—C6—C5	−179.3 (4)	Br4—C17—C18—C13	178.8 (3)
C2—C1—C6—C5	−0.1 (5)	C12—C13—C18—C17	−177.4 (4)
O1—C1—C6—C7	−2.2 (6)	C14—C13—C18—C17	1.2 (6)
C2—C1—C6—C7	177.0 (3)	C6—C7—N1—C8	−178.4 (4)
C5—C6—C7—N1	177.4 (4)	C9—C8—N1—C7	−120.1 (5)
C1—C6—C7—N1	0.2 (6)	C11—C10'—N2—C9	−101.3 (11)
N1—C8—C9—N2	72.0 (5)	C11—C10'—N2—C10	−50.4 (9)
N2—C10—C11—C10'	−47.6 (8)	C8—C9—N2—C10'	136.5 (8)
N2—C10—C11—N3	60.4 (10)	C8—C9—N2—C10	103.6 (6)
N2—C10'—C11—C10	57.2 (10)	C11—C10—N2—C10'	53.8 (10)
N2—C10'—C11—N3	−36.2 (12)	C11—C10—N2—C9	−158.1 (7)
N3—C12—C13—C18	−178.6 (4)	C13—C12—N3—C11	−175.4 (5)
N3—C12—C13—C14	2.9 (6)	C10—C11—N3—C12	158.2 (7)
C18—C13—C14—O2	−179.6 (3)	C10'—C11—N3—C12	−153.8 (7)

### Hydrogen-bond geometry (Å, °)

**Table:**

*D*—H···*A*	*D*—H	H···*A*	*D*···*A*	*D*—H···*A*
N1—H1A···O1	0.97 (6)	1.70 (6)	2.553 (5)	144 (5)
N3—H3A···O2	0.87 (6)	1.84 (6)	2.597 (4)	144 (5)
